# Postoperative dynamics of inflammatory markers in patients with uncomplicated recovery after colorectal cancer surgery: a preliminary inflammatory benchmarks study

**DOI:** 10.1038/s41598-026-59229-9

**Published:** 2026-06-28

**Authors:** Mohammed. N. Abdelaziz, Mohammed. Y. Eldomyaty

**Affiliations:** Faculty of Medicine, Mansoura University, Mansoura, Egypt

**Keywords:** Colorectal cancer, Inflammatory markers, Neutrophil-to-lymphocyte ratio, Platelet-to-lymphocyte ratio, Systemic immune-inflammation index, Reference values, Uncomplicated recovery, Cancer, Diseases, Gastroenterology, Medical research, Oncology

## Abstract

Colorectal cancer (CRC) remains a leading cause of cancer-related morbidity and mortality worldwide, with surgical resection as the cornerstone of curative therapy for localized disease. Surgery triggers a profound systemic inflammatory response that influences recovery, complications, and oncologic outcomes. While elevated NLR, PLR, and SII pre- or postoperatively predict adverse events like anastomotic leakage and poor survival, their normal temporal trajectories in uncomplicated cases remain poorly defined, hindering clinical interpretation. This preliminary study was conducted at two tertiary centers, screening 88 consecutive adults (≥ 18 years) undergoing elective curative (R0) CRC resection (stages I–III, open/laparoscopic). Inclusion required Clavien-Dindo grade ≤ I recovery (no intervention beyond symptomatic treatment; ERAS discharge criteria met) and complete blood counts (preop baseline ≤ 7 days, POD1/15/30). Exclusions: emergencies, immunosuppression, severe comorbidities (e.g., eGFR < 30, HbA1c > 10), neoadjuvant < 4 weeks prior, grade ≥ II complications (e.g., leak, sepsis, readmission). Only the uncomplicated cohort (*n* = 47; median age 50 years; 62% female) was finally analyzed. EDTA-blood analyzed via automated hematology (neutrophils/lymphocytes/platelets); markers calculated; trajectories via Friedman test; 5th–95th percentile intervals. All markers declined significantly (*P* < 0.001). NLR medians: POD1 12.5 (IQR 5.6–26.1), POD15 3.4 (2.5–6.4), POD30 2.6 (1.7–5.1); 95th percentiles 49.0 to 11.9. PLR: POD1 366.1 (223–682), POD15 244.9 (173–330), POD30 194.5 (129–319); 95th 1455 to 658.5. SII: POD1 3520 (1473–8806), POD15 1244 (757–2223), POD30 789 (470–1389); 95th 17,063 to 3311. Neutrophils fell (8.99 to 4.4 × 10^9/L), lymphocytes rose (0.71 to 1.25 × 10^9/L). Uncomplicated CRC resection recovery features rapid normalization of NLR, PLR, and SII over 30 days, providing preliminary time-specific percentile-based benchmarks for describing expected postoperative inflammatory trajectories. These findings should be interpreted as exploratory and require validation in larger multicenter cohorts before clinical application.

## Introduction

Colorectal cancer (CRC) is among the most frequently diagnosed malignancies worldwide and remains a leading cause of cancer-related morbidity and mortality despite advances in screening and multimodal treatment^1,2^. Surgical resection is the mainstay of curative-intent therapy for localized disease; however, it elicits a profound and tightly regulated systemic inflammatory and immune response that can influence postoperative recovery, the development of complications, and long-term oncologic outcomes^3,4^. In this context, there is growing interest in simple blood count–derived inflammatory indices that may capture the balance between pro-tumor inflammation and anti-tumor immunity in the perioperative period^1,5^.

Among these indices, the neutrophil-to-lymphocyte ratio (NLR), platelet-to-lymphocyte ratio (PLR), and systemic immune-inflammation index (SII; platelets × neutrophils/lymphocytes) have emerged as inexpensive and widely available biomarkers that integrate information from routine complete blood counts^5^. Elevated values of NLR, PLR, and SII have been consistently associated with adverse oncologic outcomes, including poorer overall and disease-free survival in CRC, as demonstrated in recent meta-analyses and large cohort studies^5–7^. Furthermore, several perioperative studies suggest that increased preoperative or early postoperative levels of these markers are linked to a higher risk of postoperative complications, particularly infectious complications and anastomotic leakage, after colorectal surgery^1,3,4,8^.

Despite this accumulating evidence, most available data focus on prognostic or predictive thresholds derived from heterogeneous populations that include patients at varying stages of disease, with different treatment modalities, and with different postoperative courses^2,3,9^. Importantly, prior work has often relied on single preoperative or postoperative measurements, even though these indices are inherently dynamic and likely to vary substantially in response to surgical trauma, perioperative hemodynamic changes, and evolving immune homeostasis^1,2^. The normal postoperative trajectory and time-dependent reference values of SII, NLR, and PLR in patients experiencing an uncomplicated recovery after colorectal cancer resection remain poorly defined, limiting their interpretability when used for early detection of complications in individual patients^1,2,4^.

Characterizing the expected postoperative patterns of these markers in a well-defined cohort with uneventful recovery could provide a crucial framework for distinguishing physiological inflammatory responses from early deviations suggestive of occult complications. For example, prior studies indicate that NLR typically rises sharply on the first postoperative day after colorectal resection, yet only persistently elevated or excessively high values appear to correlate with subsequent adverse events^4,8^. Similarly, SII and PLR may increase in the immediate postoperative period, but their prognostic significance likely depends on both absolute levels and temporal evolution^1,3,10^. Establishing robust, time-specific reference ranges in uncomplicated cases is therefore essential before these indices can be reliably applied as decision-support tools in routine postoperative monitoring.

Accordingly, the primary aim of the present study was to describe postoperative trajectories and generate preliminary percentile-based benchmarks for SII, NLR, and PLR. By providing detailed serial measurements in this low-risk reference population, this work seeks to offer clinically useful benchmarks against which deviations in individual patients may be interpreted, thereby refining the early identification of pathologic postoperative inflammation and supporting more timely intervention^1,2,4^.

## Methods

### Setting and study design

This preliminary study was conducted from September 2024 to July 2025 at two tertiary centers, including Mansoura Gastrointestinal Surgical Center and Mansoura Oncology Surgical Center. Eighty-eight participants participated in this study. The study included patients with histopathologically confirmed colorectal carcinoma, colon or rectum, amenable to surgical resection at any TNM stage. The patients included in this study were those who underwent elective colorectal cancer surgery, either the open method or the laparoscopic method, for definitive cures. This study employed a prospective design to establish reference intervals for systemic inflammatory markers. Participants were screened and enrolled consecutively upon admission, following a predefined research protocol designed to capture standard inflammatory trajectories.

### Selection of study participants

Consecutive patients presenting for elective colorectal cancer resection were evaluated for study eligibility. Patient recruitment occurred during the preoperative assessment period (1–7 days before surgery), with written informed consent obtained before surgical intervention. Potential participants were identified through the surgical oncology service, and initial screening was performed by trained research coordinators using prospectively developed inclusion and exclusion criteria. Patients aged ≥ 18 years undergoing elective colorectal cancer resection with curative intent via any surgical approach are eligible. Essential to inclusion is achievement of uncomplicated postoperative recovery, defined as Clavien-Dindo Grade I or less (no complications requiring intervention beyond symptomatic treatment) and successful discharge meeting ERAS criteria (adequate pain control on oral analgesia, restored vital signs, independent mobilization, tolerance of oral diet, restored bowel function). Participants must have a prospective collection of complete blood count with serial postoperative measurements. Complete demographic, surgical, and 30-day postoperative documentation must be available.

Exclusion applies to patients aged < 18 years, those unable to consent, pregnant/lactating women, or those on immunosuppressive therapy (corticosteroids ≥ 10 mg/day, biologics, calcineurin inhibitors). Tumor-related exclusions include peritoneal carcinomatosis, non-curative resection (R1/R2), and non-adenocarcinoma histology. Surgical exclusions encompass emergency/urgent procedures, inflammatory bowel disease, familial adenomatous polyposis, reoperation for recurrence, intraoperative conversion due to major complications, or blood loss > 1500 mL. Any Clavien-Dindo Grade II or higher complication—including anastomotic leak, abscess, sepsis, unplanned ICU admission, transfusion ≥ 2 units, or readmission within 30 days—mandates exclusion. Laboratory exclusions include preoperative hemoglobin < 8 g/dL, platelets < 50 or > 1000 × 10⁹/L, WBC < 2.0 or > 20 × 10⁹/L, or < 50% completeness of serial samples. Severe comorbidities (eGFR < 30, Child-Pugh Class C cirrhosis, uncontrolled diabetes HbA1c > 10%, NYHA Class IV heart failure, COPD requiring home oxygen, recent MI/stroke within 6 months) are excluded. Neoadjuvant therapy completed < 4 weeks pre-surgery or active systemic infection within 7 days of surgery also results in exclusion.

### Data collection and laboratory data

Baseline demographic information was collected via a structured questionnaire at enrollment, including age, sex, body mass index (BMI, calculated as weight in kilograms divided by height in meters squared), and relevant medical and surgical history. Baseline laboratory parameters were obtained within 7 days before surgery, including complete blood count with differential. Operative data were documented prospectively, including operative time (minutes, from skin incision to skin closure), blood loss (estimated), type of resection performed (right hemicolectomy, left hemicolectomy, anterior resection, abdominoperineal resection, or subtotal colectomy), surgical approach (open, laparoscopic, robotic), conversion status if applicable, and intraoperative complications. Perioperative anesthetic and hemodynamic data were extracted from operative records. Neoadjuvant therapy (yes/no, type, completion date) and planned adjuvant chemotherapy (yes/no) were documented.

### Postoperative follow-up and complications assessment

Postoperative hospital course was documented daily through discharge, including vital signs, pain scores (Visual Analog Scale), oral intake tolerance, bowel function recovery (documented timing of first flatus and first stool), and any adverse events. All postoperative events and complications were classified using the Clavien-Dindo Classification System (Grades 0–V) by a physician researcher blinded to inflammatory marker results. Discharge summaries, operative notes, and hospital records were reviewed to confirm uncomplicated recovery status and absence of Grade II or higher complications. Patients were contacted at 30 days postoperatively via telephone to assess for any additional complications, readmission, or emergency department visits not reflected in hospital records.

### Blood sample collection and processing

#### Collection protocol and timing

Venous blood samples were obtained prospectively 24 h postoperatively (POD1), days 15 and 30, for complete blood count analysis and calculation of NLR, PLR, and SII. Blood samples were collected into EDTA (ethylenediaminetetraacetic acid) tubes (lavender-topped tubes, 2.7 mL or 3 mL volume) for complete blood count and differential analysis. Samples were collected before intravenous medication administration when feasible. A fasting state was not required for postoperative samples, given acute postoperative metabolic alterations; however, samples were obtained at standardized times to minimize diurnal variation in white blood cell differentials.

#### Sample handling and analysis

Immediately after collection, blood samples were labeled with two patient identifiers (name and medical record number), collection date and time, and tube type. Samples were gently inverted 8–10 times to ensure adequate mixing with anticoagulant but were not shaken vigorously to prevent hemolysis. Transportation to the laboratory occurred within 30 min of collection at room temperature (20–24 °C). Samples were not refrigerated before processing to avoid temperature-induced alterations in cell counts. Complete blood count and five-part white blood cell differential (neutrophils, lymphocytes, monocytes, eosinophils, basophils) were performed using automated hematology analyzers [specify analyzer model and manufacturer: e.g., Sysmex XN-9100, Beckman Coulter DxH 900]. Instrument calibration and quality control were performed daily according to manufacturer specifications. Samples were analyzed within 2 h of collection. The analyzer measured absolute counts (cells/µL) for neutrophils, lymphocytes, monocytes, and platelets. If an automated differential was flagged as abnormal by the analyzer (e.g., immature cells, atypical lymphocytes), manual 100-cell differential review was performed by a certified laboratory technologist. Hemolyzed, clotted, or lipemic samples were rejected, and recollection was requested within the protocol window when feasible. All results were recorded directly into a secure electronic laboratory information system.

### Statistical analysis

All statistical analyses were performed using IBM SPSS Statistics for Windows, version 27.0 (IBM Corp., Armonk, NY, USA). A two-sided P-value < 0.05 was considered statistically significant. Continuous variables are presented as medians with interquartile ranges (IQRs) due to non-normal distributions (assessed by the Shapiro–Wilk test and visual inspection of histograms/Q–Q plots). Categorical variables are expressed as frequencies and percentages. The study included 88 patients who underwent curative resection for colorectal cancer and had complete serial blood sampling on postoperative day 1 (POD1), day 15, and day 30. Patients were classified into two groups based on the occurrence of 30-day postoperative complications according to the Clavien–Dindo classification (any grade ≥ II was considered a complication). Because the primary aim of the final analysis was to describe the normal postoperative trajectory in patients with uncomplicated recovery, the main results focus on the subgroup of patients without complications (*n* = 47). Baseline demographic and clinical characteristics of this uncomplicated subgroup are summarized using descriptive statistics only. The postoperative course of three systemic inflammatory markers was evaluated in patients with uncomplicated recovery:

- Neutrophil-to-Lymphocyte Ratio (NLR) = absolute neutrophil count / absolute lymphocyte count.
- Platelet-to-Lymphocyte Ratio (PLR) = platelet count / absolute lymphocyte count.
- Systemic Immune-Inflammation Index (SII) = (platelet count × absolute neutrophil count) / absolute lymphocyte count.

Values of NLR, PLR, and SII were calculated at three time points: POD1, day 15, and day 30.

To describe the overall trajectory (change over time) within the uncomplicated group, the Friedman test (non-parametric equivalent of repeated measures ANOVA) was applied to each marker separately. This test assesses whether there is a statistically significant change across the three related time points. Exploratory percentile distributions (5th–95th percentiles) were calculated to provide preliminary benchmarks of expected inflammatory marker behavior in uncomplicated recovery rather than formal clinical reference intervals. Percentiles were calculated using the default percentile estimation method implemented in IBM SPSS Statistics (Explore procedure), which is based on the empirical distribution of the observed data with interpolation between adjacent ordered observations when required. These percentile values are intended to represent the expected range of inflammatory marker levels during normal postoperative recovery after colorectal cancer surgery. The calculation of exploratory percentile-based benchmarks was based on this prospectively tracked cohort to ensure that the benchmarks reflect a controlled, ‘normal’ recovery environment rather than retrospective clinical convenience. All analyses were performed on complete cases (patients with available data at all three time points). No imputation was required for missing data in the uncomplicated subgroup used for the primary analysis.

## Results

Of the 88 patients who underwent curative resection for colorectal cancer during the study period with complete serial blood sampling, 47 patients (53.4%) experienced uncomplicated postoperative recovery. The baseline demographic, clinical, tumor, and operative characteristics of the uncomplicated group are presented in Table 1. The study cohort comprised patients with a median age of 50 years (IQR: 44–64 years), with a female predominance of 61.7%. 87.2% of patients were nonsmokers. Regarding medication history, antihypertensive drugs were the most commonly used medication class (29.8%), followed by oral hypoglycemic agents (17%) and statins (10.6%). Hypertension was the most frequent comorbidity, affecting 29.8% of patients, followed by diabetes mellitus in 19.1%. A family history of colorectal cancer was documented in 70.2% of patients. Previous gastrointestinal tract surgery was reported in 80.9% of patients. Regarding tumor characteristics, the majority of patients presented with stage T3 (76.6%). The predominant nodal staging was N0 (48.9%). Overall staging distribution showed stage I tumors in 6.4% of patients, stage II in 42.6%, and stage III in 46.8%. The cecum and ascending colon were the most common anatomical location (27.7%), followed by the sigmoid colon (21.3%). The median tumor length was 6 cm (IQR, 4.5–8 cm), with a median thickness of 25 mm (IQR, 15–40 mm). Histopathologically, moderately differentiated adenocarcinoma predominated (76.6%), with poorly-differentiated tumors in 17% and well-differentiated tumors in 6.4%. Regarding operative characteristics, anterior resection (31.9%) and right hemicolectomy (29.8%) were the most frequently performed procedures. The median operative duration was 180 min (IQR, 150–240 min). Median intraoperative blood loss was 200 ml (IQR, 100–400 ml). Three patients (6.4%) received intraoperative blood transfusions. The median body mass index (BMI) was 28 kg/m² (IQR, 24.2–32.1 kg/m²), and the median preoperative carcinoembryonic antigen (CEA) level was 5.4 ng/ml (IQR, 1.9–11.2 ng/ml).

**Table 1:** Baseline demographic, clinical, and tumor characteristics of patients with uncomplicated postoperative recovery.

Parameter	Uncomplicated cases *N*, %/median, IQR
Number of participants	47
Age (years)	50 (44–64)
Gender	
Females	29 (61.7%)
Males	18 (38.3%)
Smoking status	
Nonsmoker	41 (87.2%)
Current smoker	6 (12.8%)
Drug history	
Oral hypoglycemic drugs	8 (17%)
Statins	5 (10.6%)
Antihypertensive drugs	14 (29.8%)
Aspirin	4 (8.5%)
Comorbidities	
Hypertension	14 (29.8%)
Diabetes	9 (19.1%)
Cardiac disease	4 (8.5%)
Liver cirrhosis	1 (2.1%)
Family history of CRC	33 (70.2%)
History of GIT surgery	38 (80.9%)
Warning signs	
Unexplained anemia	20 (42.6%)
Nocturnal symptoms	6 (12.8%)
Onset of age > 50 years	19 (40.4%)
Unintended weight loss	34 (72.3%)
Bloody stool	15 (31.9%)
Greasy stool	3 (6.4%)
Bleeding per rectum	17 (36.2%)
Persistent vomiting	10 (21.3%)
TNM stage	
Tumor	
T1	0 (0%)
T2	2 (4.3%)
T3	36 (76.6%)
T4	9 (19.1%)
Node	
N0	23 (48.9%)
N1	20 (42.6%)
N2	4 (8.5%)
N3	0 (0%)
N4	0 (0%)
Metastasis	
Yes	2 (4.3%)
Overall staging	
Stage I	3 (6.4%)
Stage II	20 (42.6%)
Stage III	22 (46.8%)
Stage IV	2 (4.2%)
Tumor characteristics	
Anatomical location	
Cecum and Ascending colon	13 (27.7%)
Transverse colon	2 (4.2%)
Descending colon	6 (12.8%)
Sigmoid	7 (14.9%)
Rectosigmoid	10 (21.3%)
Rectum	9 (19.1%)
Tumor length (cm)	6 (4.5-8)
Tumor thickness (mm)	25 (15–40)
Histopathological grade	
Well differentiated	3 (6.4%)
Moderate differentiated	36 (76.6%)
Poorly differentiated	8 (17%)
Operation characteristics	
Operation nature	
Rt hemicolectomy	14 (29.8%)
Extended Rt hemicolectomy	1 (2.1%)
Lt hemicolectomy	5 (10.6%)
Sigmidectomy	5 (10.6%)
Ant resection	15 (31.9%)
Proctectomy	1 (2.1%)
Total colectomy	1 (2.1%)
Abdominoperineal resection	5 (10.6%)
Operation type	
Laparoscopic	15 (31.9%)
Open	32 (68.1%)
Operation duration (minutes)	180 (150–240)
Blood loss (ml)	200 (100–400)
Intraoperative Blood Transfusion	
Yes	3 (6.4%)
BMI (kg/m^2^)	28 (24.2–32.1)
Preoperative CEA (ng/ml)	5.4 (1.9–11.2)

Data are presented as numbers and percentages (N, %) for categorical variables and medians with interquartile ranges (IQR) for continuous variables. CRC: colorectal cancer; GIT: gastrointestinal tract; TNM: tumor, node, metastasis staging system; cm: centimeters; mm: millimeters; Rt: right; Lt: left; Ant: anterior; BMI: body mass index; CEA: carcinoembryonic antigen.

Longitudinal hematological parameters during the postoperative period are summarized in Table 2. Hemoglobin levels decreased from a median of 10.89 g/dl (IQR, 9.8–12.2 g/dl) on postoperative day 1 to 10.15 g/dl (IQR, 9.4–11.6 g/dl) on day 15, with a slight increase to 10.4 g/dl (IQR, 9.8–11.8 g/dl) by day 30. Platelet counts remained relatively stable, ranging from 295.5 k/µl (IQR, 220–391 k/µl) on day 1 to 311.8 k/µl (IQR, 218–423 k/µl) on day 15, and decreasing slightly to 288 k/µl (IQR, 252.1–320.4 k/µl) on day 30. Neutrophil counts demonstrated a pronounced decline over the postoperative period, decreasing from 8.99 k/µl (IQR, 7.4–12.62 k/µl) on day 1 to 4.7 k/µl (IQR, 3.9–6.8 k/µl) on day 15 and further declining to 4.4 k/µl (IQR, 3.2–5 k/µl) by day 30. Conversely, lymphocyte counts increased from 0.71 k/µl (IQR, 0.52–1.65 k/µl) on day 1 to 1.4 k/µl (IQR, 0.95–1.7 k/µl) on day 15, with a slight decrease to 1.25 k/µl (IQR, 0.8–2.1 k/µl) on day 30. Total white blood cell (WBC) counts followed a similar pattern to neutrophils, declining from 10.35 k/µl (IQR, 8.4–14.5 k/µl) on day 1 to 7.4 k/µl (IQR, 6.04–9.5 k/µl) on day 15 and remaining stable at 7.2 k/µl (IQR, 5.8–8.7 k/µl) on day 30. Red cell distribution width (RDW) increased slightly from 14.8% (IQR, 14.1–21.4%) on day 1 to 16.6% (IQR, 14.1–19.6%) on day 15 and 16.8% (IQR, 14.7–17.9%) on day 30.

**Table 2:** Blood counts and hematological parameters in the uncomplicated recovery group across the thirty-day postoperative period.

Parameters	Day (1)	Day (15)	Day (30)
Hg (g/dl)	10.89 (9.8–12.2)	10.15 (9.4–11.6)	10.4 (9.8–11.8)
Platelets (k/ul)	295.5 (220–391)	311.8 (218–423)	288 (252.1-320.4)
Neutrophils (k/ul)	8.99 (7.4-12.62)	4.7 (3.9–6.8)	4.4 (3.2-5)
Lymphocytes (k/ul)	0.71 (0.52–1.65)	1.4 (0.95–1.7)	1.25 (0.8–2.1)
WBCs (k/ul)	10.35 (8.4–14.5)	7.4 (6.04–9.5)	7.2 (5.8–87)
RDW (%)	14.8 (14.1–21.4)	16.6 (14.1–19.6)	16.8 (14.7–17.9)

Longitudinal changes in hematological parameters were measured on postoperative day 1 (POD1), approximately 24 h after surgery, day 15, and day 30 in 47 patients with uncomplicated recovery. Data are presented as medians with interquartile ranges (IQR). Hg: hemoglobin; WBC: white blood cells; RDW: red cell distribution width.

All three systemic inflammatory markers demonstrated statistically significant decreases across the postoperative period (Table 3). The platelet-to-lymphocyte ratio (PLR) declined from a median of 366.1 (IQR, 223–681.6) on day 1 to 244.9 (IQR, 173.1–330.1) on day 15, and further decreased to 194.5 (IQR, 128.5–318.7) on day 30 (*P* < 0.001). The neutrophil-to-lymphocyte ratio (NLR) similarly decreased from 12.5 (IQR, 5.6–26.1) on day 1 to 3.4 (IQR, 2.5–6.4) on day 15 and reached 2.6 (IQR, 1.7–5.1) by day 30 (*P* < 0.001). The systemic immune-inflammation index (SII) demonstrated the most pronounced decline, decreasing from 3520.3 (IQR, 1473–8805.8) on day 1 to 1243.7 (IQR, 757–2222.6) on day 15 and 789 (IQR, 470–1389.1) on day 30 (*P* < 0.001). Statistical significance for all trends was confirmed using the Friedman test.

**Table 3:** Systemic inflammatory markers across the postoperative period in uncomplicated recovery.

Parameters	Day (1)	Day (15)	Day (30)	*P*-trend
Platelet-to-lymphocyte ratio	366.1 (223-681.6)	244.9 (173.1-330.1)	194.5 (128.5-318.7)	< 0.001*
Neutrophil-to-lymphocyte ratio	12.5 (5.6–26.1)	3.4 (2.5–6.4)	2.6 (1.7–5.1)	< 0.001*
SII index	3520.3 (1473–8805.8)	1243.7 (757–2222.6)	789 (470–1389.1)	< 0.001*

SII: systemic immune-inflammation index; p-trend from Friedman test (non-parametric repeated measures ANOVA equivalent).

**Fig. 1:**
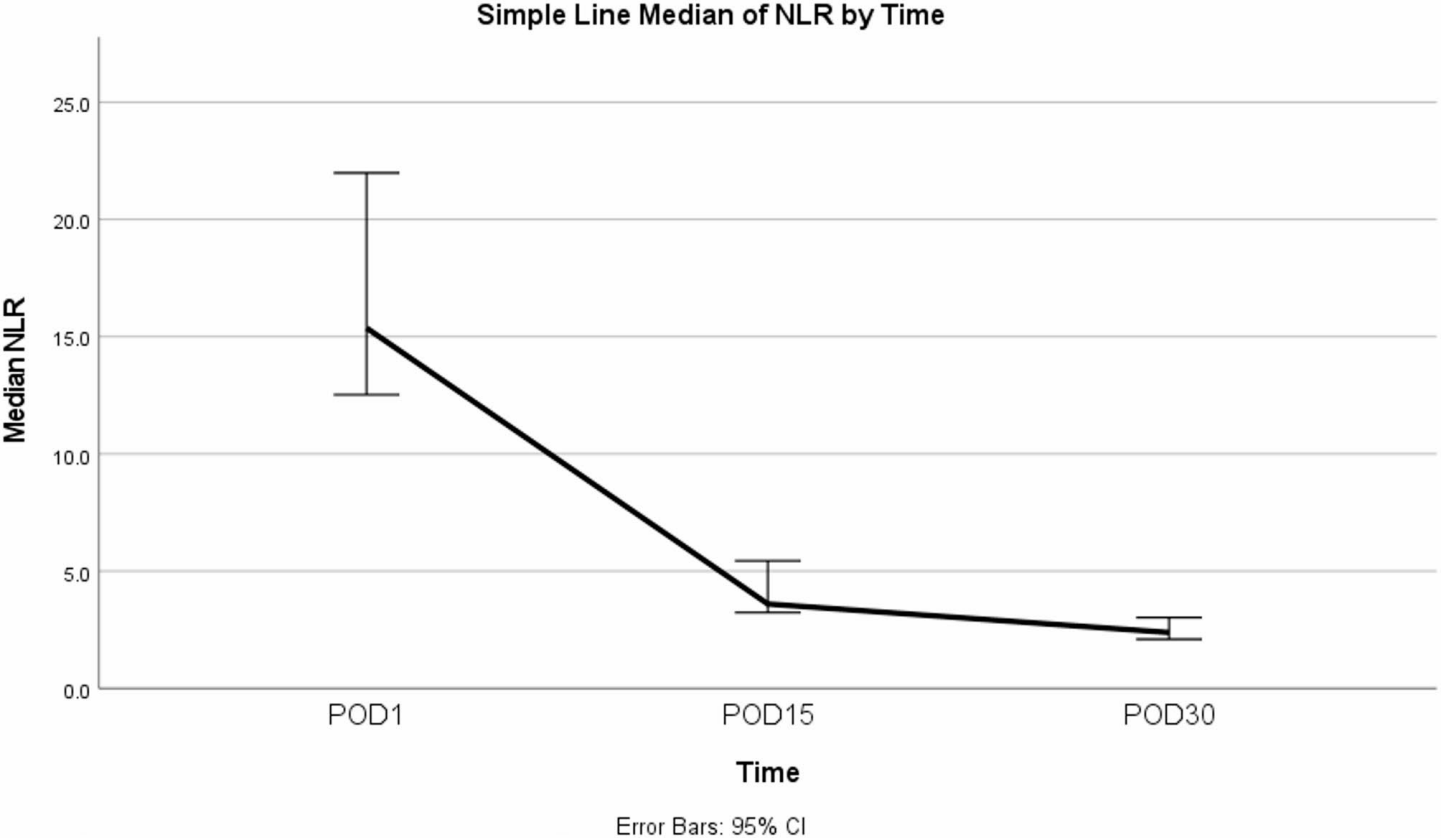
Temporal changes in median neutrophil-to-lymphocyte ratio (NLR) across postoperative time points (POD1, POD15, and POD30). The median NLR demonstrated a progressive decline over time, with error bars representing 95% confidence intervals.

**Fig. 2:**
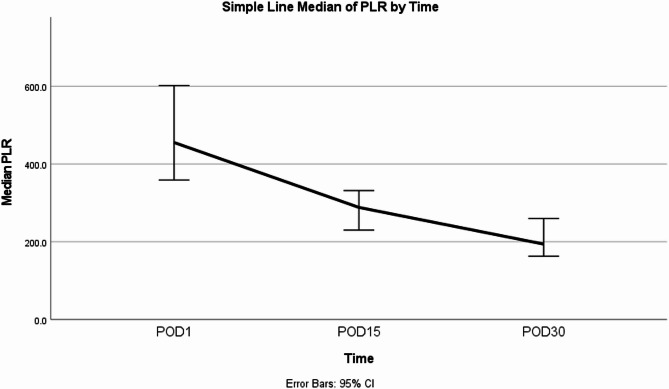
Temporal changes in median platelet-to-lymphocyte ratio (PLR) across postoperative time points (POD1, POD15, and POD30). Median PLR values gradually decreased during follow-up. Error bars indicate 95% confidence intervals.

**Fig. 3:**
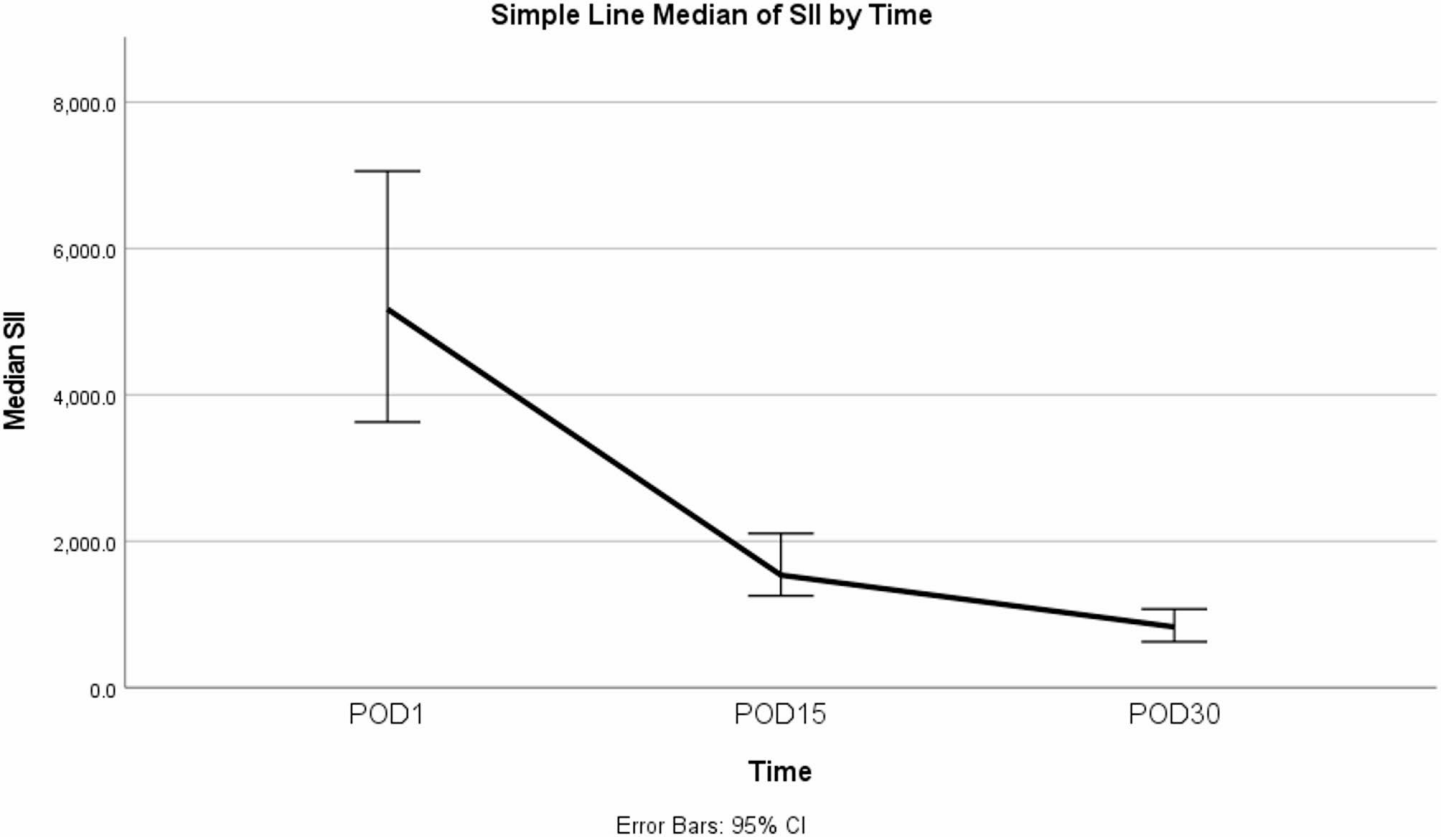
Temporal changes in median systemic immune-inflammation index (SII) across postoperative time points (POD1, POD15, and POD30). Median SII values showed a marked reduction over time, with 95% confidence intervals displayed as error bars.

Reference intervals for systemic inflammatory markers during normal postoperative recovery are provided in Table 4. For PLR, the 5th and 95th percentiles on day 1 were 117.23 and 1455.03, respectively; on day 15, these values narrowed to 88.19 and 675.52; and by day 30, they ranged from 88.39 to 658.50. For NLR, the 5th and 95th percentiles on day 1 were 1.32 and 49.01, respectively; on day 15, these values decreased to 2.09 and 14.13; and on day 30, they ranged from 0.78 to 11.91. For the SII index, the 5th and 95th percentiles on day 1 were 341.94 and 17063.31, respectively; on day 15, these narrowed to 454.98 and 6593.04; and by day 30, they ranged from 240.23 to 3311.25. Additional percentile values (10th, 25th, 50th [median], and 75th) for each marker at all three time points are summarized in Table 4. Figures 1 and 2, and 3 illustrate the time course of markers (NLR, PLR, and SII) postoperatively.

**Table 4:** Preliminary percentile-based benchmarks for systemic inflammatory markers during uncomplicated postoperative recovery.

Marker	5th	10th	25th	50th	75th	90th	95th
PLR							
Day 1	117.23	153.61	223	366.06	681.58	1128.66	1455.03
Day 15	88.19	109.43	173.08	244.90	330.13	474.13	675.52
Day 30	88.39	103.38	128.54	194.53	318.68	534.56	658.50
NLR							
Day 1	1.32	2.95	5.56	12.54	26.11	36.25	49.01
Day 15	2.09	2.19	2.45	3.40	6.42	12.14	14.13
Day 30	0.78	0.92	1.67	2.57	5.09	8.91	11.91
SII index							
Day 1	341.94	728.99	1472.96	3520.26	8805.79	13747.1	17063.31
Day 15	454.98	600.63	756.96	1243.69	2222.56	3227.93	6593.04
Day 30	240.23	348.16	469.98	788.97	1389.09	2889.91	3311.25

## Discussion

This study demonstrates that systemic inflammatory markers—neutrophil-to-lymphocyte ratio (NLR), platelet-to-lymphocyte ratio (PLR), and systemic immune-inflammation index (SII)—follow a consistent and predictable temporal trajectory in patients experiencing uncomplicated postoperative recovery following curative colorectal cancer resection. All three indices showed statistically significant declines over the 30-day postoperative period, with the most pronounced change occurring between postoperative day 1 and day 15. Specifically, NLR declined from a median of 12.5 to 2.6 (*P* < 0.001), PLR decreased from 366.1 to 194.5 (*P* < 0.001), and SII fell from 3520.3 to 789 (*P* < 0.001). These findings provide contemporary evidence that successful surgical recovery in colorectal cancer patients is characterized by rapid normalization of systemic inflammation, a process that can be reliably monitored through accessible peripheral blood parameters.

The marked elevation of inflammatory indices on postoperative day 1, immediately following surgical resection, is consistent with evidence showing that the magnitude of the postoperative stress response varies by surgical approach and extent of tissue trauma, as illustrated by the TaLaR trial on inflammatory stress responses after transanal versus laparoscopic total mesorectal excision^11^. This initial surge is consistent with established pathophysiology: major surgery triggers a robust systemic inflammatory response characterized by activation of neutrophils and platelets in excess of lymphocyte proliferation, resulting in elevated NLR, PLR, and SII values in the immediate postoperative period^12–14^. The subsequent dramatic decline in these indices by day 15, with further reduction by day 30, aligns with the anticipated kinetics of surgical recovery in cancer-free patients. A systematic review evaluating 19 studies involving 7,023 colorectal cancer patients found that early postoperative NLR values were predictive of anastomotic leakage and other complications, supporting the clinical utility of these temporal dynamics in risk stratification^12^. Furthermore, in a recent investigation of 200 colorectal cancer patients, postoperative NLR, PLR, and SII values on postoperative days 1 and 6 significantly correlated with length of hospital stay, suggesting that inflammatory kinetics are sensitive indicators of recovery trajectory^1^. The normalization pattern observed in our cohort is also mechanistically aligned with cellular immune recovery: the reduction in absolute neutrophil counts coupled with increases in lymphocyte counts—reflecting resolution of the acute stress-induced lymphopenia—drives the decline in these ratio-based indices, representing the transition from innate immune dominance to restored adaptive immunity.

The distinction between preoperative and postoperative inflammation is a critical insight supported by contemporary evidence. Longitudinal studies in stage III colorectal cancer patients have shown that postoperative inflammation-based markers, rather than preoperative values, more accurately predict long-term oncological outcomes^15^. In this context, postoperative normalization of NLR, CAR (C-reactive protein-to-albumin ratio), and lymphocyte-to-monocyte ratio (LMR) was associated with significantly better overall survival (hazard ratio 0.49 for normalized NLR; 95% CI 0.27–0.90) and recurrence-free survival compared with persistent elevation^15^. Moreover, this prognostic advantage of normalized postoperative inflammation was independent of tumor stage and adjuvant chemotherapy, suggesting that the host inflammatory state—rather than tumor biology alone—influences survival outcomes. Our findings complement this literature by providing preliminary benchmark distributions that describe expected postoperative inflammatory trajectories in uncomplicated recovery. The percentile distributions provided (5th to 95th percentiles for NLR, PLR, and SII at each time point) enable clinical identification of patients whose inflammatory response deviates from expected norms, potentially indicating complications or underlying immune dysfunction.

The prognostic value of systemic inflammatory markers extends beyond the immediate postoperative period to predict late complications and long-term outcomes. Preoperative inflammatory markers have been shown to correlate with lymph node metastasis, postoperative pneumonia, and distant metastasis in colorectal cancer populations^16,17^. A 2024 meta-analysis examining the clinical significance of blood cell-derived inflammatory markers in colorectal cancer found that preoperative NLR cutoff values between 2.21 and 4.0 were significantly associated with severe complications, intra-abdominal infection, and anastomotic leakage in 13 of 19 included studies (68.4%)^12^. Additionally, in a cohort of 303 colorectal cancer patients, elevated preoperative NLR and PLR independently predicted postoperative lung metastasis and worse progression-free survival, with prognostic nutritional index (PNI) emerging as particularly sensitive (AUC 0.706) for detecting short-term postoperative complications^17,18^. The systemic immune-inflammation index, which integrates neutrophil, platelet, and lymphocyte counts, has been validated as a superior prognostic marker compared with individual ratios in several cancer types^19,20^. In gastric and breast cancer cohorts, low preoperative SII was associated with improved disease-free survival and favorable response to immunotherapy, highlighting the comprehensive nature of this index in reflecting both immune competence and tumor-promoting inflammation^19,20^.

Although the observed inflammatory trajectories provide insight into postoperative recovery dynamics, the selected sampling intervals (POD1, POD15, and POD30) may not fully capture the early postoperative period during which most clinically actionable decisions occur, particularly under ERAS pathways. Consequently, these findings should be interpreted primarily as descriptive recovery benchmarks rather than tools for immediate postoperative prediction.

### Clinical implications

The clinical and public health implications of these findings are substantial. First, the preliminary benchmark distributions generated in this study may provide contextual guidance for interpreting postoperative inflammatory recovery patterns; however, they should not be used as definitive clinical thresholds. Given the simplicity and cost-effectiveness of calculating NLR, PLR, and SII from standard complete blood count results, serial monitoring at established time points (postoperative days 1, 15, and 30) may facilitate early identification of patients at risk for complications without additional diagnostic burden or expense^1,21^. Second, these biomarkers may complement existing prognostic frameworks—such as TNM staging and Glasgow prognostic score—to enable more nuanced risk stratification and individualized treatment decisions, particularly regarding the need for intensified adjuvant therapy or enhanced surveillance protocols. Third, the observed inflammatory trajectories provide a physiological baseline against which patient-specific recovery can be evaluated; deviations from expected normalization patterns may warrant investigation for occult complications such as anastomotic leak, abscess, or infection^12,13^. Enhanced recovery after surgery (ERAS) protocols have been shown to mitigate excessive postoperative inflammation (evidenced by reduced CRP and IL-6 levels) and correlate with expedited recovery and fewer complications, suggesting that systematic monitoring of inflammatory indices could support protocol compliance and identify patients requiring intervention^22^.

### Limitations and recommendations

Several limitations merit acknowledgment. First, this investigation focused exclusively on patients experiencing uncomplicated recovery, restricting the generalizability of the reference intervals to heterogeneous surgical populations. The study did not include a comparative analysis of inflammatory trajectories in patients who experienced postoperative complications, limiting the discriminatory ability to establish threshold values for detecting aberrant recovery. Second, the relatively modest sample size (*n* = 47) and single-center design may restrict the applicability of the reference percentiles to diverse populations with varying genetic backgrounds, comorbidities, and surgical practices. Additionally, although percentile distributions were generated, the sample size does not satisfy conventional laboratory recommendations for formal reference interval establishment. Therefore, these estimates should be interpreted as preliminary benchmarks intended for hypothesis generation and future validation rather than definitive clinical reference standards. Third, the study evaluated only three systemic inflammatory markers; integration of additional biomarkers such as C-reactive protein, interleukin-6, or procalcitonin might provide complementary prognostic information and strengthen risk prediction models. Fourth, the short postoperative follow-up period (30 days) precludes assessment of the relationship between inflammatory kinetics and late complications or long-term oncological outcomes, such as recurrence-free survival or overall survival. Finally, the cross-sectional percentile analysis does not account for individual-level variability in baseline inflammatory status, preoperative comorbidities, or operative factors that may modulate the inflammatory response trajectory. Future research should prioritize prospective, multi-center investigations comparing inflammatory marker trajectories between uncomplicated and complicated recovery cohorts to establish diagnostic thresholds with optimized sensitivity and specificity for detecting postoperative morbidity. Longitudinal follow-up extending to 6–12 months postoperatively, with assessment of disease recurrence and survival outcomes, would clarify the relationship between early postoperative inflammatory normalization and long-term prognosis. Additionally, mechanistic studies integrating flow cytometry, immunophenotyping, and circulating cytokine analysis could elucidate the cellular and molecular basis of the observed inflammatory trajectories and identify patient subgroups susceptible to aberrant recovery patterns. Finally, prospective studies examining whether serial monitoring of inflammatory markers and protocol-driven interventions (such as nutritional optimization, physical rehabilitation, or targeted anti-inflammatory therapy) to promote inflammatory normalization can improve postoperative outcomes and long-term survival would strengthen the clinical utility of these accessible biomarkers. Integration of machine learning algorithms to develop personalized inflammatory recovery predictions—incorporating patient age, comorbidities, tumor stage, operative technique, and postoperative inflammatory kinetics—may further enhance clinical decision-making and support individualized postoperative management strategies. Additionally, the relatively wide spacing between postoperative measurements limited characterization of early inflammatory kinetics within the first postoperative week, which may represent the most clinically informative period for complication detection and postoperative decision-making.

## Conclusion

This study shows that NLR, PLR, and SII follow a consistent, rapidly declining trajectory over the first 30 postoperative days in colorectal cancer patients with uncomplicated recovery, accompanied by falling neutrophil and WBC counts and partial restoration of lymphocyte counts. The provision of percentile-based preliminary benchmarks at defined postoperative time points may help describe expected inflammatory recovery patterns; however, these findings should not be interpreted as definitive clinical reference ranges and require validation in larger multicenter cohorts before routine clinical use. Given that systemic inflammatory markers are inexpensive, widely available, and easily derived from routine blood counts, their serial assessment may support early recognition of deviations from expected recovery, refine postoperative risk stratification, and inform surveillance strategies in colorectal cancer care.

## Data Availability

The relevant authors of the current study have kindly provided the data sets under analysis upon appropriate demand from the corresponding author.
